# The Protective Effect of N-Acetylcysteine on Ionizing Radiation Induced Ovarian Failure and Loss of Ovarian Reserve in Female Mouse

**DOI:** 10.1155/2017/4176170

**Published:** 2017-05-21

**Authors:** Wei Gao, Jin-Xiao Liang, Chi Ma, Jing-yin Dong, Qiu Yan

**Affiliations:** ^1^Department of Biochemistry and Molecular Biology, Dalian Medical University, Dalian, China; ^2^Department of Clinical Medicine, Zhejiang University City College School of Medicine, Hangzhou, China; ^3^Department of Thoracic Surgery, Zhejiang Cancer Hospital, Hangzhou, China; ^4^Department of Surgery, The First Affiliated Hospital of Dalian Medical University, Dalian, China

## Abstract

Ionizing radiation may cause irreversible ovarian failure, which, therefore, calls for an effective radioprotective reagent. The aim of the present study was to evaluate the potential radioprotective effect of N-acetylcysteine (NAC) on ionizing radiation induced ovarian failure and loss of ovarian reserve in mice. Kun-Ming mice were either exposed to X-irradiation (4 Gy), once, and/or treated with NAC (300 mg/kg), once daily for 7 days before X-irradiation. We examined the serum circulating hormone levels and the development of ovarian follicles as well as apoptosis, cell proliferation, and oxidative stress 24 hours after X-irradiation. In addition, morphological observations on the endometrial luminal epithelium and the fertility assessment were performed. We found that NAC successfully restored the ovarian and uterine function, enhanced the embryo implantation, improved the follicle development, and altered the abnormal hormone levels through reducing the oxidative stress and apoptosis level in granulosa cells while promoting the proliferation of granulosa cells. In conclusion, the radioprotective effect of NAC on mice ovary from X-irradiation was assessed, and our results suggested that NAC can be a potential radioprotector which is capable of preventing the ovarian failure occurrence and restoring the ovarian reserve.

## 1. Introduction

Ovarian failure is defined as loss of ovarian physiological function and is the most serious gynecological disease that results in infertility and comes with low estradiol levels and elevated follicle-stimulating hormone (FSH) [1]. Ovarian failure occurred with the depletion of the follicles and increased rate of oocyte atresia [2]. Anti-Mullerian hormone (AMH), which is produced in the granulosa cells in late prenatal and small antral follicles, is considered as a sensitive indicator of the longitudinal decline of ovarian reserve [3]. The level of AMH decreases with age, especially after age of 30 years, where it declines more steeply [4, 5]. The ovarian granulosa cells play a key role in fertility and pregnancy by regulating ovulation and luteal regression [6]. The proliferation and apoptotic state of the ovarian granulosa cells reflect the ovarian function. Among all the physicochemical factors, ionizing radiation is the most common cause for ovarian failure which affects the fertility tremendously [7].

Ionizing radiation, including gamma and X-ray, is considered as one of the important etiologies for infertility [8]. An increasing risk of infertility and abortion was found in the pregnant women who work in nuclear industry [9]. Women who received pelvic radiation during childhood were at high risk of acute ovary failure and/or premature menopause [10]. Adult females who received radiotherapy are at high risk of oocyte loss and reduction in follicle store [11]. In addition, the impairment of ovaries induced by ionizing radiation was shown by in vivo observation [12].

Searching for more effective radioprotectors has drawn wide attention for the women of child-bearing age who may be exposed to ionizing radiation (such as radiotherapy). Ionizing radiation impairs the cellular function mainly by generating reactive oxygen species (ROS) [13, 14]. ROS plays an important role in chemical toxicants induced growth inhibition and apoptosis in cultured mouse antral follicles [15]. NAC, a well-known thiol-containing antioxidant, is a safe drug which has been wildly used in clinics for over 50 years [1]. As a precursor of intracellular cysteine [16], NAC showed its capability of facilitating intracellular glutathione (GSH) biosynthesis in different cells lines and organs. NAC administration prevented the UV exposure induced oxidative damage in mice skin [17]. Mansour and colleagues found that pretreating the rat with NAC protected the liver from gamma irradiation by increasing the antioxidative enzymes while the levels of MDA, NO(x), and DNA damage decreased [18]. In Wu and colleagues' in vitro study, N-acetylcysteine amide (NACA), a thiol antioxidant which affects the cells in an extremely similar manner to NAC, was found to attenuate radiation induced cytotoxicity in Chinese hamster ovary cells [19]. The protective effect of NAC on mouse ovary heterotopic autotransplantation was testified to promote the follicular survival and function in mice ovarian grafts [20]. Till now, the protective effect of NAC on radiation induced damage in organs (such as liver and skin) has been studied in recent years [21, 22]. However, the underlying mechanism of how NAC administration protects the ovarian function from being impaired by ionizing radiation exposure still needs further exploration.

## 2. Materials and Methods

### 2.1. Mice and NAC Treatment/Pretreatment and X-Irradiation

All experimental procedures involved in the mouse studies were approved by the Institutional Review Board in Dalian Medical University. We established the mouse POF model by using outbreed Kun-Ming mice (6 weeks old) weighing 20 ± 1 g, which were provided by Lab Animal Center in Dalian Medical University of China. After the stabilization period, all of the mice were randomly divided into 4 groups, respectively: control, X-irradiation, NAC treatment alone, and NAC/X-irradiation. Mice of X-irradiation and NAC/X-irradiation groups received whole-body X-irradiation as a single dose of 4 Gy. In NAC and NAC/X-irradiation groups, the mice were injected intraperitoneally daily with NAC (300 mg/kg, dissolved in normal saline) for 7 days before sample collection. In NAC/X-irradiation group, the mice were then exposed to a single dose of (4 Gy) whole-body X-irradiation.

X-irradiation was performed at the intensity of 100 cGy/min with a CLINAC 2300C/D-SN 27 X-ray unit.

### 2.2. Fertility Assessment

Another 24 female mice of all groups (6 mice per group) were used, mated with sexually experienced male mice at the ratio of 2 : 1 after superovulation. A vaginal plug was used to mark the first day of pregnancy (D1). The uteruses were obtained on D7.

### 2.3. ELISA

Serum was collected by centrifugation at 3000 ×g for 15 min and kept at −80°C until being assayed for estradiol, progesterone, FSH, and AMH. Four ELISA kits (Mouse Estradiol(E2)/Progesterone/FSH/AMH ELISA Kit) (LengTon, China) were used for detection of serum estradiol, progesterone, FSH, and AMH level according to the manufacturer's instructions.

### 2.4. Histopathological Examination

The ovaries or uterus was fixed in 10% formalin overnight and embedded in paraffin. Serial sections of 7 *μ*m thickness were stained with hematoxylin and eosin for light microscopic histological examination. In all ovarian samples, the eighth cut was chosen for counting the number of follicles and evaluating follicular development. Images were captured using a light microscope (Olympus, Japan). Follicles were classified as primordial, preantral, antral, and atretic as described in Britt and colleagues' study [23].

### 2.5. Western Blot

Mice were sacrificed after X-irradiation, and ovaries were removed and lysed in 6 ml 1x DPBS buffer containing 1% triton-x100, protease inhibitors, and 2 mM PMSF. After briefly homogenizing, sonicating, and centrifuging at 4°C for 30 min, the supernatant was collected, and 50 *μ*g of the lysate of each group was loaded onto a SDS-PAGE and transferred onto NC membranes and revealed with anti-PCNA rabbit monoclonal antibody (1 : 1000) (Proteintech, USA). The relative densitometric analysis of the western blot was performed using Image Pro Plus software 6.0 and expressed as a percentage of control.

### 2.6. Scanning Electron Microscopy of Pinopod

After NAC and/or X-irradiation treatment, superovulation was induced in female mice administering 5 IU of pregnant mares' serum gonadotropin intraperitoneally, followed by 5 IU human chorionic gonadotropin intraperitoneally 48 h later. Then they were placed with males: each 2 female mice were caged with one male mouse. All mice of the study groups were sacrificed on day 7 after superovulation (the pinopod only emerged during the peri-implantation period), implanted embryos in each uterine horn were counted and analyzed, and the endometrium was examined by using full-thickness tissue collected from the uterine horns of mice from all groups. For scanning electron microscopy (SEM) observation, protocol was followed as described in previous study [24]. The images were taken in scanning electron microscope (JEM-2000 EX).

### 2.7. GPx Activity Assessment

To determine the glutathione peroxidase (GPx) activity, Cellular Glutathione Peroxidase Assay Kit (Beyotime, China) was used following the manufacturer's instructions. The results were expressed as mU/mg protein.

### 2.8. Immunohistochemistry

The ovaries were harvested, fixed, and embedded in paraffin, using anti-8-OHdG mouse monoclonal antibody (1 : 100) (Santa Cruz, USA), anti-anti-cytochrome c rabbit polyclonal antibody (1 : 100) (Bioworld, USA), anti-caspase 3 rabbit polyclonal antibody (1 : 100) (Bioworld, USA), anti-PCNA polyclonal antibody (1 : 100) (Proteintech, USA), and Histostain-Plus Kits (ZSGB-BIO, China). Following deparaffinization, the tissue sections were incubated with first antibody at 4°C overnight and then reacted with biotinylated secondary anti-mouse IgG antibody for 30 min at room temperature. Streptavidin was added and followed by diaminobenzidine (DAB) staining. Subsequently, the tissue sections were counterstained with hematoxylin and images were captured on microscope (Olympus, Japan). The immunohistochemical staining was quantified by using Image Pro Plus software 6.0 and expressed as mean density.

### 2.9. Statistical Analysis

All data were presented as mean ± standard deviation (SD). The data were analyzed by one-way analysis of variance (ANOVA) and Student's *t*-test, using SPSS 11.5 software. The level of significance was set at *p* < 0.05 for all statistical analyses.

## 3. Results

### 3.1. Ovarian Weight Changes

Weights of ovaries were compared after normalization to 1 g body weight.  Table 1 showed significant reduction in ovarian weight in irradiated group compared with that of the control group. Pretreatment of NAC before X-irradiation maintained the ovarian weight to a normal level. Mice treated with NAC alone did not show any significant difference from that of the control group.

### 3.2. Circulating Hormone Levels

**Figure 1:**
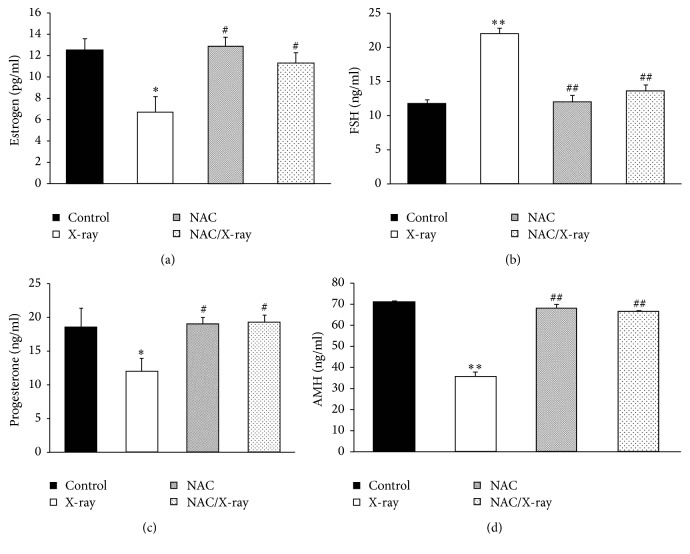
Hormone levels. Changes in serum levels of estradiol (a), FSH (b), progesterone (c) and AMH (d). Values are given as mean ± SD (^*∗*^*p* < 0.05 and  ^*∗∗*^*p* < 0.01 versus control; ^#^*p* < 0.05 and  ^##^*p* < 0.01 versus X-irradiation group).

Serum estradiol level of X irradiated group showed significant decrease to 53.89% of control values (6.71 ± 1.42 versus 12.48 ± 2.08 in control), as shown in Figure 1(a). In contrast, the serum level of FSH was significantly increased 1.9-fold (21.97 ± 0.87 versus 11.83 ± 2.10 in control) by exposure to X-irradiation, as shown in Figure 1(b). Meantime, pretreating the mice with NAC before X-irradiation restored the serum level of estradiol to nonradiation treated levels (Figure 1(a)) and the FSH level was significantly decreased in comparison to X-ray treated mice (Figure 1(b)). Also, the serum progesterone (12.03 ± 2.94 versus 18.52 ± 3.84 in control) and AMH (36.00 ± 1.69 versus 71.18 ± 1.10 in control) level were significantly decreased when mice were exposed to the X-irradiation and pretreatment with NAC significantly restored the serum level of progesterone and AMH to the control level (Figures 1(c) and 1(d)).

### 3.3. Folliculogenesis

**Figure 2:**
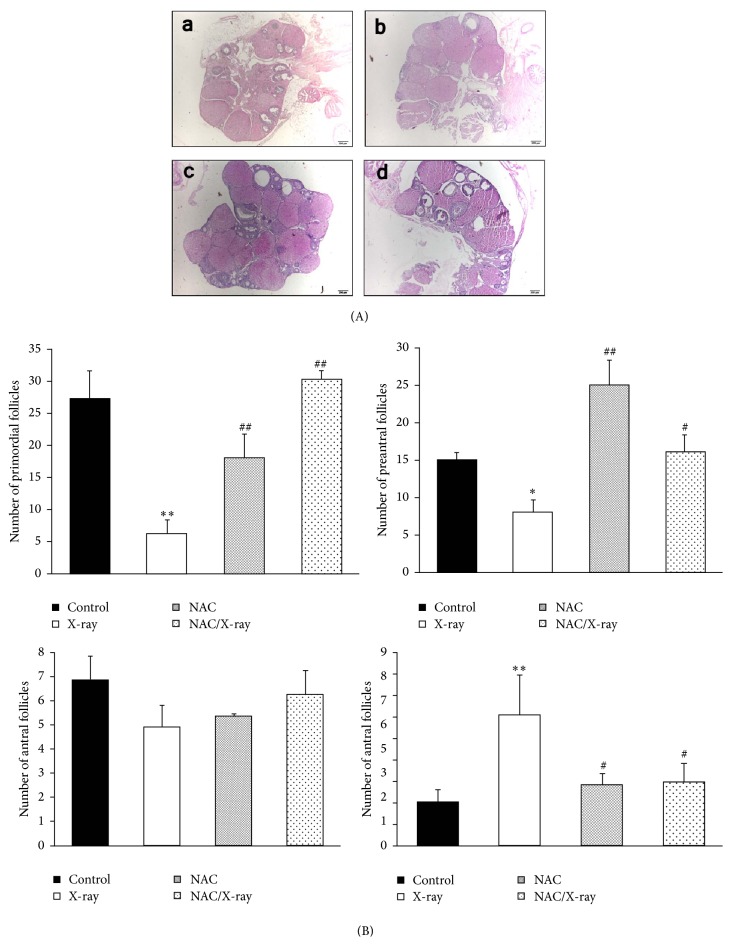
Representative HE stained ovarian tissue sections and morphometric analysis of ovarian follicle populations. (A) Representative photomicrographs of hematoxylin and eosin-stained ovarian tissue sections. Histological sections of control (a) and NAC treated group (c) represent similar organization, with number of follicles in different stages. In contrast, drastic decrease of follicle number was shown in the X-irradiation group. Sections taken from the mice pretreated with NAC before X-irradiation showed near-normal organization, with number of growing follicles. Scale bar, 200 *μ*m. (B) Numbers of (a) primordial, (b) preantral, (c) antral, and (d) atretic ovarian follicles. Each bar represents mean ± SD of at least three independent experiments (^*∗*^*p* < 0.05 and  ^*∗∗*^*p* < 0.01 versus control; ^#^*p* < 0.05 and  ^##^*p* < 0.01 versus X-irradiation group).

The population of primordial follicles was reduced by 81% in irradiated ovaries (6.14 ± 2.2 versus 27 ± 4.5 in controls) (Figures 2(A)-(a) and 2(B)). Also, as shown in Figures 2(A)-(b) and 2(B), X-irradiation reduced the number of preantral follicles by 39% (7.99 ± 1.53 versus 14.91 ± 1.04 in controls). However, there was no significant difference in number of antral follicles in the X-irradiation group compared with control group (Figures 2(A)-(c) and 2(B)). Moreover, we found significantly increasing number of atretic follicles in irradiated ovaries by 300% (6.05 ± 2.89 versus 2.02 ± 1.10 in controls). Meantime, we found that pretreatment with NAC before X-irradiation significantly increased the number of primordial and preantral follicles and reduced the number of the atretic follicles compared to the X-irradiation group. No significant effect was found on the number of antral follicles compared with the control group.

### 3.4. Immunohistochemical Analysis and Expression of PCNA

Immunohistochemical detection of PCNA was assessed to evaluate the ovarian granular cells proliferation. Strong expression of PCNA was shown in the granular cells of the ovaries obtained from the mice of control and NAC treated groups (Figures 4(A)–4(a) and (c)) while significantly weak expression of PCNA was shown in the X-irradiation group (Figure 4(A)-(b)). Meantime, pretreatment of NAC to the mice significantly augmented the PCNA expression to normal level (Figure 4(A)-(d)). The immunohistochemical staining was quantified as shown in Figure 4(B) and expressed as mean density.

We also assessed the PCNA expression by western blot (Figures 4(C) and 4(D)). Ovarian tissue obtained from the X irradiated mice showed significantly lower expression compared with control group, and treating with NAC before X-irradiation significantly increased the PCNA expression. The relative densitometric analysis of the western blot was given in Figure 4(D).

### 3.5. Oxidative Stress

The oxidative stress and activity of antioxidant enzymes were assessed by immunohistochemical detection of 8-OHdG formation and GPx activity assay. As shown in Figure 5(A), in the ovaries of control and NAC group, the cells showed weak positivity of 8-OHdG (Figures 5(A)–5(a) and (c)), and high 8-OHdG formation was observed in X-irradiation group (Figure 5(A)-(b)). Meantime, pretreating the mice with NAC protected the ovary from X-irradiation induced 8-OHdG formation (Figure 5(A)-(d)). The immunohistochemical staining of 8-OHdG was quantified as mean density and the results are represented in Figure 5(B).

As shown in Figure 5(C), X-irradiation stimulated significant reduction of GPx activity in ovary tissue by 43% compared to the control group, while pretreating the mice with NAC significantly restored the GPx activity.

### 3.6. Apoptosis

The immunohistochemical analysis of cytochrome c and caspase 3 were conducted to assess the state of apoptosis in mice ovary (Figures 6(A)–6(D)). We found low expression of cytochrome c (Figures 6(A)–6(a) and (c)) and caspase 3 (Figure 6(B)-(a), (c)) in ovarian granulosa and theca interstitial cells obtained from the mice treated without or with NAC alone. In contrast, we found significantly increased level of cytochrome c (Figure 6(A)-(b)) and caspase 3 (Figure 6(B)-(b)) in the samples obtained from the mice treated with X-irradiation alone compared with those of the control group. Treating the mice with NAC before X-irradiation significantly decreased the expression of cytochrome c (Figure 6(A)-(d)) and caspase 3 (Figure 6(B)-(d)). The immunohistochemical staining of cytochrome c and caspase 3 was quantified as mean density and the results are represented in Figures 6(C) and 6(D).

We also assessed the state of apoptosis by TUNEL assay (data not shown). Widespread TUNEL signal was visible in ovary tissue obtained from mice exposed to X-irradiation while little fluorescence signal was observed in ovaries of the mice without or with NAC treatment alone. Meantime, significantly weakened TUNEL signal was observed in the ovary of NAC pretreated mice.

### 3.7. Morphological Change in Uterus and Fertility Assessment

**Figure 3:**
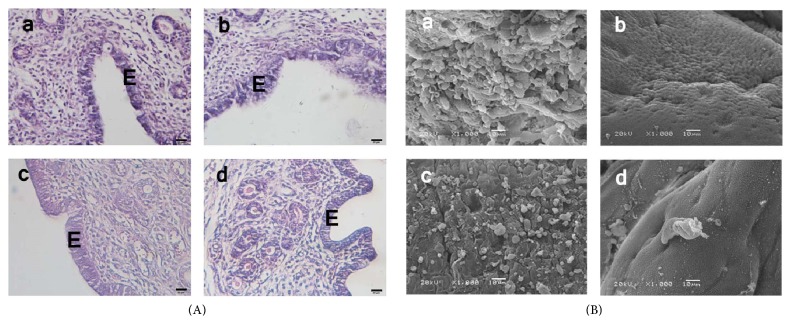
Morphological changes and fertility assessment. (A) Representative HE staining. Sections obtained from the uterine of the mice of control (a) and NAC treated (c) groups showed normal endometrial luminal epithelial structure, while the sections of the X-irradiation group (b) showed high degeneration of the endometrial luminal epithelium with vacuole appearance. Sections of the NAC pretreatment group (d) showed regeneration of luminal epithelium of normal structure. Scale bar, 20 *μ*m. E: endometrial luminal epithelial structure. (B) SEM observation of mouse endometrium. In control (a) and NAC treatment (c) group, multiple of round smooth-surfaced projects were found, which is called pinopod. X-irradiation exposure almost completely terminated the pinopod formation as shown in (b). In the NAC pretreatment group (d), pinopod formation was restored represented as blebs. Scale bar, 10 *μ*m.

**Figure 4:**
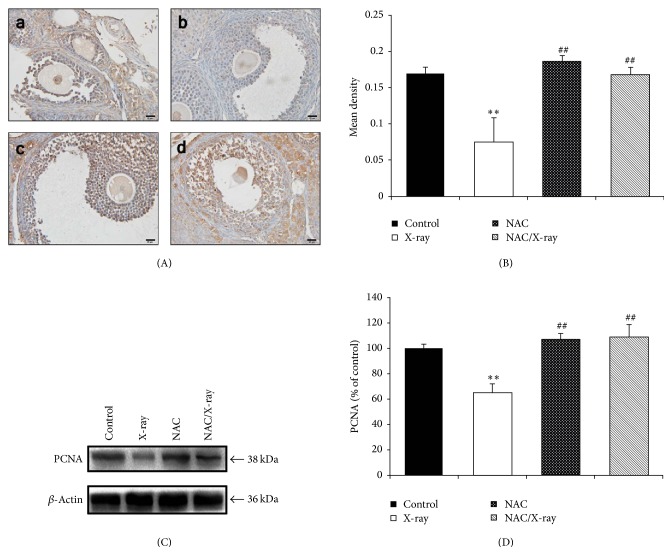
Proliferation state of follicles. (A) Immunohistochemical localization of PCNA. Expression of PCNA in control (a) and NAC treated group (c) was at similar level, and sections from the mice exposed to X-irradiation represented weak expression of PCNA (b). PCNA expression of the NAC pretreatment group showed similar level to the control group (d). Scale bar, 20 *μ*m. (B) Quantitative image analysis for IHC staining expressed as mean density in each group. Each column represents the mean ± SD of at least three independent experiments (^*∗∗*^*p* < 0.01 versus control; ^##^*p* < 0.01 versus X-irradiation group). (C) Detection of PCNA by western blot images was representative of at least three independent experiments. (D) Quantitative image analysis for western blot. Each column represents the mean ± SD of at least three independent experiments (^*∗∗*^*p* < 0.01 versus control; ^##^*p* < 0.01 versus X-irradiation group).

**Figure 5:**
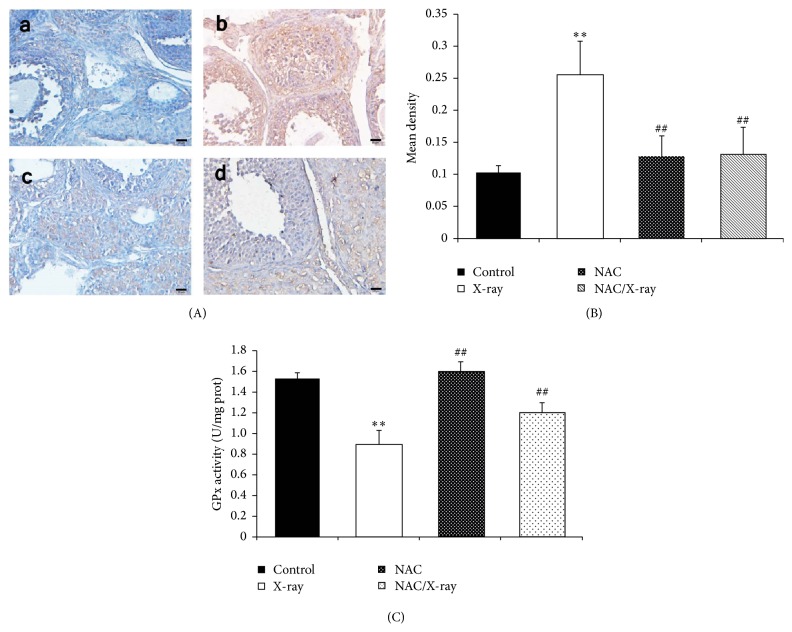
Oxidative stress analysis. (A) Immunohistochemical localization of 8-OHdG in ovary tissue. Expression of 8-OHdG showed a minimum degree in control (a) and NAC treatment group (c), while, in X-irradiation group (b), the 8-OHdG formation was highly increased, and pretreatment of NAC limited the 8-OHdG expression (d). Scale bar, 20 *μ*m. (B) Quantitative image analysis for IHC staining expressed as mean density in each group. Each column represents the mean ± SD of at least three independent experiments (^*∗∗*^*p* < 0.01 versus control; ^##^*p* < 0.01 versus X-irradiation group). (C) GPx activity level in wet ovary tissue was represented as mU/mg of protein. X-irradiation significantly decreased the GPx activity concentration. Each column represents the mean ± SD of at least three independent experiments. (^*∗∗*^*p* < 0.01 versus control; ^##^*p* < 0. versus X-irradiation group).

**Figure 6:**
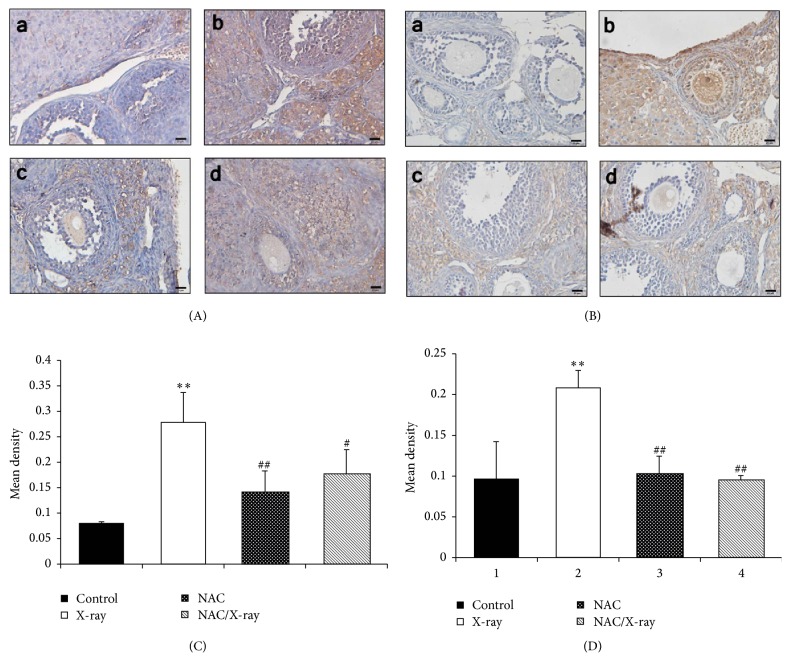
Apoptosis analysis in ovarian granule cells. (A) Immunohistochemical localization of cytochrome c. (a, b) Sections in control and NAC treatment group showed weak cytochrome c expression. (c) X-irradiation increased the cytochrome c expression. (d) NAC pretreatment limited the cytochrome c formation. Scale bar, 20 *μ*m. (B) Immunohistochemical localization of caspase 3. (a, b) Sections in control and NAC treatment group showed minimum degree of caspase 3 expression. (c) X-irradiation increased the caspase 3 expression. (d) NAC pretreatment limited the caspase 3 expression. Scale bar, 20 *μ*m. (C, D) Quantitative image analysis for IHC staining of cytochrome c and caspase 3, respectively, expressed as mean density in each group. Each column represents the mean ± SD of at least three independent experiments. (^*∗∗*^*p* < 0.01 versus control; ^##^*p* < 0.01 versus X-irradiation group).

Uterine sections obtained from control and NAC treated groups showed normal histological structure of endometrial luminal epithelial structure and the underlying glandular structure (Figures 3(A)–3(a) and (c)). After X-irradiation, the endometrial luminal epithelial structure showed degeneration structure with blebbing membrane (Figure 3(A)-(b)). Interestingly, the tissue obtained from the mice pretreated with NAC showed normal histological structure of endometrial luminal epithelial structure (Figure 3(A)-(d)).

Pinopodes, coming with the loss of microvilli on the surface of endometrial epithelium, are large, rounded, and smooth-surfaced projections of the apical plasma membrane. By SEM, uterine endometrium obtained from the mice of control and NAC treated groups showed uniformly distributed pinopode presenting cells (Figures 3(B)–3(a) and (c)). After X-irradiation, the endometrium represent no pinopod formation at all (Figure 3(B)-(b)), and the endometrium obtained from the mice of NAC pretreated group showed blebs (Figure 3(B)-(d)), which is considered an initial phase of pinopod maturation [24].

The protective effects of NAC on the low reproductive capacity in terms of fecundability and fecundity induced by X-irradiation were shown in Table 2. Four in six female mice of control group were mated and pregnant with a litter size of 18.2 embryos/female. Only one in six irradiated mice was mated and yet not pregnant (0% versus 100% in control) with a litter size of 0 (0 versus 18.2, embryos for controls). Treating the mice with NAC before X-irradiation preserved their fertility and increased the ability of females to become pregnant (100%) with litter size of 10 pups/female. Female mice treated with NAC only were led to (100%) pregnancy with litter size of 20.6 embryos/female.

## 4. Discussion

Ovarian failure is characterized by loss of follicles and folliculogenesis due to low proliferation of ovarian granular cell. Our morphological detection showed that X-irradiation significantly promoted the depletion of the primordial follicles. This result was basically consistent with Mandl's study, which had shown that the primordial follicles were much more sensitive than the antral follicles to the ionizing radiation exposure [25]. In the present study, NAC administration effectively preserved the primordial follicles' reserve and promoted the maturation of follicle. The proliferation of granulosa cells plays an important role in follicles development and maturation. It has been reported that ionizing radiation decreased the proliferation of the granulosa cells in rat ovary [12]. In rat ovaries, PCNA was not detected in granulosa cells or oocytes in primordial follicles but was highly expressed with the initiation of follicle growth [26]. Our result coincided with the report that, in control group, high expression of PCNA was detected in granulosa cells of antral follicle which indicated high level of proliferation [26]. We also found that the PCNA expression was significantly increased by NAC pretreatment before X-irradiation. It has been reported that ionizing radiation elevated the p53 expression which then regulated the expression of downstream gene p21, thus leading to the downregulation of PCNA [27]. Bae and colleagues' study showed an inhibitory effect of NAC administration on p53 expression and phosphorylation [28], from which we inferred that NAC elevated the PCNA expression by downregulating the p53 expression. Our present study showed an in vivo evidence that NAC can promote granulosa cells proliferation, thus protecting the follicle development from X-irradiation.

The loss of follicles and folliculogenesis leads to disordered hormone secretion as well as the abnormal morphology and function of the ovarian effector organs (such as uterus). Ovarian failure comes with low serum levels of estradiol and elevated FSH level and leads to infertility [1]. In the present study, we found lower estradiol levels and extremely higher FSH concentration in X irradiated group, and the ovary weight was significantly reduced. Our results showed typical ovarian failure. Moreover, the AMH level was also decreased by X-irradiation exposure. AMH, which is produced in the granulosa cells in late prenatal and small antral follicles, is considered as a sensitive indicator of the longitudinal decline of ovarian reserve [3]. AMH value showed an extremely low, even undetectable level in POF (premature ovarian failure) patients [29]. Low AMH value combined with abnormal FSH level has strong impact on pregnancy rate [30]. In our study, the similar result was demonstrated, which further indicated an ovarian failure. Meantime, in NAC/X-ray group, such abnormal hormone condition was significantly recovered, which indicated that NAC is capable of protecting the ovarian function from ionizing radiation.

In addition, we found significant uterine lining mucosal epithelium degeneration and limited embryo implantation of female mice after X-irradiation exposure. In Said and colleagues' study, the degeneration of endometrial epithelium from mice was observed after exposure to ionizing radiation [12], and similar result was also obtained in Bath and colleagues' study [31]. Estradiol secretion is essential for maintaining the normal growth of endometrial epithelial structure [32]. In our results, pretreatment with NAC increased the serum estradiol level and thus inhibited the endometrial epithelium degeneration. Pinopods, as an important morphologic marker of uterine function, are surface projections of the endometrial cells involved in uterine pinocytosis in mammals [33]. The presence and development of the pinopods depend on the ovarian secretion of progesterone [34] which is closely associated with normal luteal cells function and follicular development. X-irradiation significantly depressed the plasma progesterone concentration in mice by promoting the luteal regression [35, 36]. We found a significant disappearance of the pinopodes on the surface of the mice endometrial epithelium after exposure to X-irradiation. Interestingly, NAC administration increased the progesterone concentration and, meantime, preserved the pinopods formation as shown in our result, which means the endometrial receptivity was restored. We also found that NAC pretreatment before X-irradiation could elevate the fertility capacity of the female mice, which was due to the increased estradiol level. Estradiol has a positive effect on sexual behavior; increase in E2 levels facilitates female mate-searching and assessment behaviors [24]. Our results suggested that NAC attenuated the deterioration of uterine and preserved the fertility capacity of female mice through normalizing the disordered circulating hormone levels induced by X-irradiation.

The central mechanism of how NAC protected the ovary from X-irradiation was the inhibition of oxidative stress. Ionizing radiation can directly disrupt the atomic structures and result in chemical and biological changes in living cells, which initiates physiological changes or cell death [14]. Also, indirectly, it can generate reactive chemical species like ROS that may damage nucleic acids, proteins, and lipids by acting through radiolysis of water [37]. When attacked by ROS, bases in DNA can be oxidized, such as the oxidation of deoxyguanosine (dG) to 8-OHdG [38], which is higher in aging oocytes [39]. Increased ROS level and decreased antioxidant enzymes activity (such as GPx) combined together to impair the progesterone secretion [40], endometrial epithelium degeneration, and follicle regression [41, 42]. NAC is a well-known sulfhydryl-containing antioxidant and its role in radioprotection has been explored in several studies [43, 44]. In vivo study has reported that NAC preserved the ovarian function in ovary tissue transplantation through scavenging oxygen free radicals [20]. On the other hand, cell proliferation can be suppressed by ROS [45]. According to our previous discussion, NAC administration increased the PCNA level in granulosa cells through reducing the ROS generation. In the current study, the antioxidant effect of NAC was shown by increasing the GPx activity and decreasing the 8-OHdG formation induced by X-irradiation.

Besides oxidative stress, we also assessed the apoptosis induced by X-irradiation in mice ovarian granular cell. Increase of ROS level leads to release of mitochondrial cytochrome c and triggering of apoptosis by activating caspase 3 [46]. The ovarian granulosa cells play a key role in fertility and pregnancy by regulating ovulation and luteal regression [6]. Cell apoptosis is closely associated with the initiation of follicular atresia and luteal cell death [47]. In the present study, the apoptosis induced by X-irradiation in ovarian granulosa cells was observed. NAC inhibits apoptosis induced by toxicant in nonovary cells [17, 48]. Also, treating the Chinese hamster ovary cells with NAC can effectively inhibit cell apoptosis induced by radiation [19]. Our study indicated that NAC protected the ovarian function from X-irradiation and enhances the follicular development through decreasing the apoptosis induced by oxidative stress.

In conclusion, NAC effectively restored the ovarian function and fertility capability of female mice and may be capable of being used as an ovarian radioprotector. However, the underlying mechanism of how NAC prevents the irradiation-induced inhibition of granulose cell proliferation still needs further exploration.

## Figures and Tables

**Table 1:** Effect of NAC injection (300 mg/kg, i.p.; once daily for 7 days) and/or whole-body X-irradiation on ovarian weights.

Groups	Ovarian weight	
	mg	mg/g body weight
Control	22.79 ± 4.87	1.26 ± 0.29
X-ray	12.54 ± 8.06^*∗∗*^	0.71 ± 1.28^*∗∗*^
NAC	23.96 ± 5.46^##^	1.19 ± 0.89^##^
NAC/X-ray	20.79 ± 4.01^##^	1.20 ± 0.28^##^

Data expressed as mean ± SD. *∗∗* or ##: significantly different from control or X-irradiation group, respectively, at *p* < 0.01 using one-way ANOVA followed by Tukey–Kramer as a post hoc test.

**Table 2:** Reproductive performance of control, irradiated, and/or NAC treated females.

Groups	Mating percentage	Fecundability	Fecundity
Control	66.7%	100%	18.2
X-ray	16.7%	0%	0^*∗∗*^
NAC	50%	100%	20.6^##^
NAC/X-ray	16.7%	100%	10^#^

Fecundability was expressed as a percentage of pregnant females among mated females. Fecundity was expressed as the number of embryos per mated females. *∗∗* or ##/#: significantly different from control or X-irradiation group, respectively, at *p* < 0.01/0.05.
