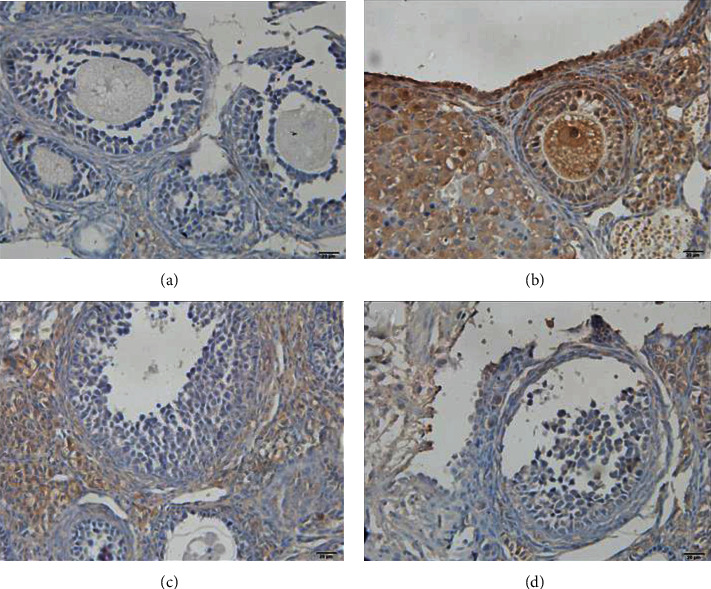# Corrigendum to “The Protective Effect of N-Acetylcysteine on Ionizing Radiation Induced Ovarian Failure and Loss of Ovarian Reserve in Female Mouse”

**DOI:** 10.1155/2021/9817842

**Published:** 2021-07-12

**Authors:** Wei Gao, Jin-Xiao Liang, Chi Ma, Jing-yin Dong, Qiu Yan

**Affiliations:** ^1^Department of Biochemistry and Molecular Biology, Dalian Medical University, Dalian, China; ^2^Department of Clinical Medicine, Zhejiang University City College School of Medicine, Hangzhou, China; ^3^Department of Thoracic Surgery, Zhejiang Cancer Hospital, Hangzhou, China; ^4^Department of Surgery, The First Affiliated Hospital of Dalian Medical Universit, Dalian, China

The article titled “The Protective Effect of N-Acetylcysteine on Ionizing Radiation Induced Ovarian Failure and Loss of Ovarian Reserve in Female Mouse” [1], contains a figure duplication issue in Figure 6(b), which was raised in a PubPeer comment [2]. Figures 6(c) and 6(d) display an area of overlap and the authors apologize for the error, which was due to the incorrect file being selected during the preparation of the figure. The correct Figure 6(b) is as follows:

## Figures and Tables

**Figure 1:**